# The mechanism of short-chain fatty acid in hypertriglyceridemic pancreatitis

**DOI:** 10.3389/fmicb.2025.1714013

**Published:** 2026-02-03

**Authors:** Tingting Li, Fuyu Deng, Jiong Xiong, Fangqi Wu, Songxun Tang, Yijie Zhang, Qiyong Zhang, Jian Song, Yi Li, Xu Liu, Yan Tang

**Affiliations:** 1 Department of Intensive Care Unit, The Affiliated Hospital of Guizhou Medical University, Guiyang, Guizhou, China; 2 Department of Specialized Ward for Pancreatitis, The Affiliated Hospital of Guizhou Medical University, Guiyang, Guizhou, China; 3 Department of Clinical Laboratory Centre, The Affiliated Hospital of Guizhou Medical University, Guiyang, Guizhou, China

**Keywords:** gut microbiota, hypertriglyceridemia pancreatitis, mechanism, short-chain fatty acid, treatment

## Abstract

**Introduction:**

The incidence of hypertriglyceridemia pancreatitis (HTGP) has been rising annually with a poor prognosis, and its pathogenesis is complex. In recent years, the role of gut microbiota and short-chain fatty acids (SCFAs) has gradually attracted attention in HTGP. Therefore, the aim of this study was to investigate the characteristic alterations in gut microbiota and SCFAs and their potential clinical significance and molecular mechanisms in HTGP.

**Materials and methods:**

This study collected feces and serum samples from 18 HTGP patients and 18 healthy volunteers. We compared baseline clinical characteristics, gut microbiota diversity (via 16S rRNA sequencing), and serum SCFA concentrations (using mass spectrometry and other techniques) between the two groups to identify characteristic alterations. Correlation analyses explored associations between microbiota, metabolites, and clinical parameters. Further animal models validated the *in vivo* functions and molecular mechanisms of SCFAs.

**Results:**

HTGP patients exhibited higher body mass index (BMI), triglyceride (TG) levels, metabolic complication incidence and disease severity scores. In addition, the gut microbiota showed significantly reduced richness and diversity in HTGP. Serum metabolomics analysis revealed significantly higher concentrations of isovaleric acid, decanoic acid and octanoic acid in HTGP patients, demonstrating good diagnostic value. Correlation analysis indicated that differential gut microbiota and metabolites were closely associated with clinical parameters. Animal experiments confirmed that butyrate can alleviate HTGP disease severity by inhibiting NF- κ B/NLRP3 pathway activation.

**Discussion:**

In summary, this study identified differential gut microbiota and SCFAs in HTGP patients compared to healthy volunteers, and confirmed that butyrate exerts a protective effect through NF-κB/NLRP3 pathway.

## 1 Introduction

Acute pancreatitis (AP) is an acute inflammatory process of the pancreas characterized by premature activation of digestive enzymes within pancreatic acinar cells, leading to self-digestion of the pancreas (Shah et al., 2009). Gallstones remain the primary cause of AP, followed by alcohol abuse (De Pretis et al., 2020; Luo et al., 2024). Due to rapid economic growth and dietary shifts, the incidence of hypertriglyceridemic pancreatitis (HTGP) has been rising globally (Simha, 2020), increasing from 14.0% in 2001 to 34.0% in 2016 (Desai et al., 2016). Compared to other forms of acute pancreatitis, HTGP is associated with more frequent and more severe complications (Li et al., 2024; Nawaz et al., 2015; Pascual et al., 2019). The management of HTGP primarily is supportive treatment (Yang et al., 2020). Moreover, there is a lack of standardized management protocols or guidelines for HTGP available both domestically and internationally. Therefore, investigating the pathophysiological mechanisms of HTGP holds significant practical importance for improving patient outcomes.

The pathogenesis of HTGP remains unclear to date. Recent studies have indicated that the gut microbiota plays a crucial role in the pathophysiology and progression of HTGP. On one hand, in the presence of HTG, the impaired intestinal barrier can increase intestinal permeability, allowing gut microbiota and their metabolites (such as endotoxins) to translocate into the bloodstream. This triggers systemic inflammatory responses, causing inflammatory damage to the pancreas. On the other hand, HTGP patients exhibit increased pathogenic bacteria and reduced beneficial bacteria in their gut microbiota. This imbalance can exacerbate the disruption of intestinal barrier function (Li G. et al., 2023). Study has demonstrated that disrupted gut microbiota correlates with poor HTGP progression in rat models (Huang et al., 2017). Gut microbiota dysbiosis activates the immune system, releasing excessive inflammatory mediators (e.g., TNF-α, IL-6). These cytokines not only exacerbate pancreatic inflammation but may also indirectly promote AP development by disrupting lipid metabolism (Qi-Xiang et al., 2022). Furthermore, gut microbiota dysbiosis in HTGP patients may lead to abnormal bile acid metabolism, which impairing lipid digestion and absorption. This exacerbating hypertriglyceridemia (Jia et al., 2018; Wang et al., 2025). Overall, gut microbiota dysbiosis, a common pathophysiological basis for hypertriglyceridemia and systemic inflammatory response, plays an important role in the pathogenesis of HTGP. However, the mechanisms by which alterations in gut microbiota structure and function influence disease severity, as well as the molecular pathways through which specific microbial communities mediate pancreatic tissue damage in HTGP patients, remain poorly understood. Although preliminary evidence has been established regarding the correlation between gut microbiota and HTGP, the profound complexity of the gut microbial ecosystem necessitates more in-depth systematic research.

Short-chain fatty acids (SCFAs) are primarily produced through microbial fermentation of undigested dietary carbohydrates in the gut, consisting mainly of acetic acid, propionic acid, and butyric acid. They serve as crucial signaling molecules for stabilizing the intestinal barrier and promoting gut immunity (Wang et al., 2012; Willemsen et al., 2003). As key metabolic products of the gut microbiota, SCFAs play a significant role in the pathogenesis of HTGP. Research indicates that SCFAs can maintain intestinal barrier function, exert anti-inflammatory effects and regulate lipid metabolism. The reduction of SCFAs of HTGP patients may disrupt lipid metabolism, further elevating serum triglyceride levels (Nogal et al., 2021). Moreover, the decreased levels of SCFAs in HTGP patients lead to increased intestinal permeability and exacerbated systemic inflammatory responses (Guo et al., 2025; Xiong et al., 2022). Assessing the level of SCFAs to understand their clinical significance may provide new theoretical insights into understanding the pathophysiological mechanisms of HTGP, while also expanding novel perspectives for clinical treatment strategies of this disease.

This study aims to systematically investigate characteristic alterations in the gut microbiota structure of HTGP patients and quantitatively analyze serum SCFAs profiles using targeted metabolomics technology. It seeks to explain the correlation between gut microbiota composition and SCFAs metabolic profiles, and evaluate the association between the level of SCFAs and clinical-pathological parameters in HTGP patients. Furthermore, this study will explore the effects of SCFAs on HTGP and their underlying mechanisms, aiming to provide novel theoretical foundations and potential therapeutic targets for HTGP prevention and treatment strategies.

## 2 Materials and methods

### 2.1 Clinical study subjects

From January 2024 to December 2024, 18 patients with hypertriglyceridemic pancreatitis (HTGP group) and 18 healthy volunteers (NC group) were recruited at Guizhou Medical University Affiliated Hospital (Guizhou Province, China). Healthy controls were enrolled based on detailed medical history, comprehensive physical examination, chest X-ray imaging, and laboratory tests including complete blood count, glucose metabolism, and hepatic/renal function. HTGP diagnosis based on the 2012 revised Atlanta criteria for AP (Banks et al., 2013). The inclusion criteria of HTGP patients is based on the 2012 revised Atlanta criteria for the diagnosis of AP. Exclusions included chronic pancreatitis, inflammatory bowel disease, cancer, irritable bowel syndrome, gastroenteritis, and patients who had used antibiotics, probiotics, or laxatives within the preceding 2 months. The study protocol strictly conducted in accordance the ethical guidelines of the Declaration of Helsinki. All subjects provided written informed consent. This study was approved by the Medical Ethics Committee of Guizhou Medical University (Approval No.: 2024 Lun Shen No. 221).

### 2.2 Preparation of fecal and serum samples

For healthy volunteers, we collected 200–300 mg of fresh fecal samples; for HTGP patients, we used sterile anal swabs to collect fresh fecal samples. All samples were placed in 5 mL sterile cryovials with clearly labeled sample numbers on the vial walls. Collected samples were immediately placed in a 4°C transport cooler and transported to the laboratory within 30 min. Upon arrival at the laboratory, samples were rapidly frozen in liquid nitrogen (1 min) before being transferred to a −80°C ultra-low temperature freezer for long-term storage. The next morning, peripheral venous blood was collected from all subjects in a fasting state. Serum was separated by centrifugation at 3,000 rpm for 10 min. A 500μL aliquot was transferred to a 1.5 mL sterile cryovial and stored in a −80°C ultra-low temperature freezer. Concurrently, clinical and pathological data were systematically collected for HTGP patients, while baseline information (including age, sex, body mass index, and other demographic characteristics) was recorded for healthy volunteers.

### 2.3 DNA collection and extraction, 16S rRNA gene sequencing

Total DNA was extracted from samples using the CTAB/SDS method. The 16S rRNA gene at different positions was amplified using the 515F-806R primers. PCR products were evaluated via 2% agarose gel electrophoresis, followed by purification using the Qiagen Gel Extraction Kit. Sequencing libraries were generated using the NEBNext^®^ Ultra™ II DNA Library Preparation Kit (Product No.: E7645) and sequenced on the Illumina NovaSeq platform.

### 2.4 Sequence data analysis

Initial amplified sequence variants (ASVs) underwent denoising and species annotation using the QIIME platform (QIIME2-202006 version). Species richness and diversity were assessed using indices including Chao1, Shannon, and Simpson. Beta diversity was evaluated based on Unifrac distances. Principal Coordinate Analysis (PCoA) was employed to further illustrate differences in species diversity among samples. LEFSe analysis (Segata et al., 2011) was performed using LEFSe software (v1.0) with LDA scores set to 4. Differences in microbial community abundance between groups were analyzed using MetaStat and *t*-tests in R software v3.5.3. Functional annotation was performed using PICRUSt2 (version 2.1.2-b) (Douglas et al., 2020). Spearman’s rank correlation analysis was employed to determine correlations between gut microbiota, short-chain fatty acids, and clinical parameters.

### 2.5 Targeted metabolomics study and data analysis

GC-MS analysis was performed using a Shimadzu GC2030-QP2020 NX GC-MS system (Alseekh et al., 2021). All instrument details and detailed methods are provided in Supplementary File.

Multivariate data containing sample names, compound names, and concentrations were analyzed using SIMCA v16.0.2 software (Umetrics, Sweden). After log transformation and centering of the data, principal component analysis (PCA) modeling was performed. Outliers in the dataset were identified using the 95% confidence interval in the PCA score plot. Data were log-transformed and normalized in UV format using SIMCA v16.0.2 software. Orthogonal Partial Least Squares-Discriminant Analysis (OPLS-DA) modeling was performed on the first principal component. Model quality was first assessed via seven-fold cross-validation; model validity was then determined using the obtained R^2^ and Q^2^ values; finally, model validity was further verified through permutation testing.

### 2.6 Animals and experimental model

Eighteen Sprague-Dawley (SD) rats (male, 4–6 weeks old, weighing 150–200 g) were purchased from Guangdong Weitong Lihua Animal Technology Co., Ltd. [License No.: SCXK(Yue) 2022-0063]. Rats were housed in SPF conditions with free access to food and water. All animals underwent 1 week of acclimation before experimentation.

SD rats were randomly assigned to three groups (*n* = 6/group): (a) HTGP Group: HTG rats were fed a high-fat, high-cholesterol diet for 4 weeks, followed by two intermittent L-arginine injections (2.5 g/kg, 1-h interval) to induce acute pancreatitis (Hegyi et al., 2004). (b) SCFAs + HTGP Group: On top of the HTG diet, rats received 100 mM butyrate solution intervention starting 4 weeks before modeling. (c) Control rats were fed a standard diet for 4 weeks and received an equivalent volume of saline (1-h interval). Twenty-four hours after L-arginine injection, rats were anesthetized with the inhalation anesthetic isoflurane (Induction concentration is 3–4%, Maintain a concentration of 2–2.5%), and blood and pancreatic tissue samples were collected for subsequent analysis.

### 2.7 Histopathology, immunohistochemistry, and immunofluorescence staining

Rat pancreatic tissue was fixed in 4% paraformaldehyde solution, followed by dehydration, embedding, sectioning, staining, and neutral resin mounting. Sections were examined microscopically and imaged. Immunohistochemical detection of anti-p65 (1:100 dilution) was performed, with quantitative analysis using ImageJ software.

For pancreatic tissue immunofluorescence, sections were dewaxed and subjected to microwave repair. Sections were blocked with 3% bovine serum albumin (BSA) for 30 min. Subsequently, sections were incubated overnight at 4°C with anti-NLRP3 (1:100, zen-bio, Cat.381207) and anti-Caspase-1 (1:100, zen-bio, Cat.341030) antibodies. Sections were washed and incubated with corresponding fluorescently conjugated secondary antibodies at room temperature for 1 h. DAPI was added for nuclear staining, incubated in the dark for 10 min. Images were captured using a fluorescence microscope.

### 2.8 Western blotting

Tissues were lysed in RIPA buffer (containing protease inhibitors, prepared fresh) to extract total protein. Protein quantification was performed using a BCA assay kit. Equal amounts of protein were separated on 8–12% SDS-PAGE gels, then transferred to polyvinylidene difluoride (PVDF) membranes and blocked with 5% non-fat milk for 30 min. Wash the blocked membrane with TBST and incubate overnight at 4°C with the primary antibody. Subsequently, wash the membrane and incubate with HRP-conjugated secondary antibody at room temperature for 2 h. Protein bands were visualized by chemiluminescence. Analysis was performed using ImageJ software.

### 2.9 ELISA assay

IL-1β, IL-18, TNF-α, and TG levels in serum were measured using an ELISA kit according to the manufacturer’s instructions.

### 2.10 Statistical analysis

Statistical analysis was performed using SPSS 26.0 software (IBM Corp., Armonk, NY, United States) and GraphPad Prism 8.0 (GraphPad Software, United States). Normality of distribution was first assessed for continuous variables. Quantitative data are presented as mean ± standard deviation (SD). Intergroup comparisons were performed using independent samples *t*-tests (for equal variances) or one-way ANOVA (for multiple comparisons). Quantitative data not normally distributed were expressed as median (interquartile range) [M (P25, P75)], and intergroup comparisons were conducted using the Mann-Whitney U test or Wilcoxon signed-rank test. Qualitative data were expressed as counts (percentages), and intergroup comparisons were performed using Pearson’s chi-square (χ^2^) test or Fisher’s exact test. A *P* < 0.05 was considered statistically significant.

## 3 Results

### 3.1 Baseline characteristics of patients

Analysis of baseline characteristics revealed that the body mass index (BMI) was significantly higher in the HTGP group than in the NC group (29.0 ± 3.0 vs. 21.2 ± 2.0, *P* < 0.001). Further analysis revealed significantly higher prevalence of obesity (55.6% vs. 5.6%, *P* = 0.003) and morbid obesity (38.9% vs. 0%, *P* = 0.008) in the HTGP group compared to the NC group. Regarding metabolic-related comorbidities, the HTGP group exhibited significantly higher prevalence of prior diabetes (66.7% vs. 0%, *P* < 0.001) and fatty liver disease (33.3% vs. 0%, *P* = 0.019) compared to the NC group. Laboratory findings showed significantly elevated serum triglyceride levels (median 39.1, IQR 21.1–49.4 vs. 1.1, IQR 0.8–1.4, *P* < 0.001) and C-reactive protein levels (median 29.4, IQR 7.1–87.5 vs. 2.2, IQR 1.9–3.2, *P* < 0.001) were significantly elevated. Regarding disease severity assessment, the HTGP group exhibited significantly higher APACHE II scores, SOFA scores, and Balthazar scores compared to the NC group (all *P* < 0.05). Detailed baseline characteristics comparison between the two groups is presented in Table 1.

**TABLE 1:** Demographic and clinical characteristics of the two groups.

Variables	HTGP group (*N* = 18)	NC group (*N* = 18)	*P-*value
Age (years), mean (SD)	38.8 (9.4)	37.4 (10.6)	0.680
Male, n (%)	10 (55.6)	10 (55.6)	1.000
BMI (kg/m^2^), mean (SD)	29.0 (3.0)	21.2 (2.0)	< 0.001
Overweight (BMI 25–29.9 kg/m^2^), n (%)	10 (55.6)	1 (5.6)	0.003
Obesity (BMI ≥ 30 kg/m^2^), n (%)	7 (38.9)	0 (0.0)	0.008
Smoking, n (%)	9 (50.0)	11 (61.1)	0.738
Drinking, n (%)	14 (77.8)	16 (88.9)	0.658
Comorbid abnormalities, n (%)			
Hypertension	5 (27.8)	2 (11.1)	0.402
Diabetes	12 (66.7)	0 (0.0)	< 0.001
Fatty liver	6 (33.3)	0 (0.0)	0.019
Laboratory examinations			
Triglyceride (mmol/L), median (IQR)	39.1 (21.1, 49.4)	1.1 (0.8, 1.4)	< 0.001
CRP (mg/L), median (IQR)	29.4 (7.1, 87.5)	2.2 (1.9, 3.2)	< 0.001
APACHE II, median (IQR)	2.0 (2.0, 2.5)	0.0 (0.0, 0.0)	< 0.001
SOFA score, median (IQR)	0.0 (0.0, 1.0)	0.0 (0.0, 0.0)	0.004
Balthazar score E, n (%)	5 (27.8)	0 (0.0)	0.045
Local complications, n (%)			
APFC	8 (44.4)	0 (0.0)	0.003
ANC	1 (5.6)	0 (0.0)	1.000
Infected necrosis	1 (5.6)	0 (0.0)	1.000
Systematic complication, n (%)			
SIRS	4 (22.2)	0 (0.0)	0.104
AKI	1(5.6)	0 (0.0)	1.000
Liver damage	5(27.8)	0 (0.0)	0.045
Outcome			
Hospital stay (days), median (IQR)	9.5 (6.8, 12.5)	0.0 (0.0, 0.0)	< 0.001

Hospital stay (days), median (IQR) 9.5 (6.8, 12.5) 0.0 (0.0, 0.0) < 0.001. BMI body mass index, CRP C-reactive protein, APACHE II Acute Physiology and Chronic Health Evaluation II, SOFA Sequential Organ Failure Assessment, APFC acute peripancreatic fluid collection, ANC acute necrotic foci accumulation, SIRS systemic inflammatory response syndrome, AKI acute kidney injury, SD standard deviation, IQR interquartile range, n number of patients.

### 3.2 Alterations in gut microbiota diversity and abundance in HTGP patients

**FIGURE 1:**
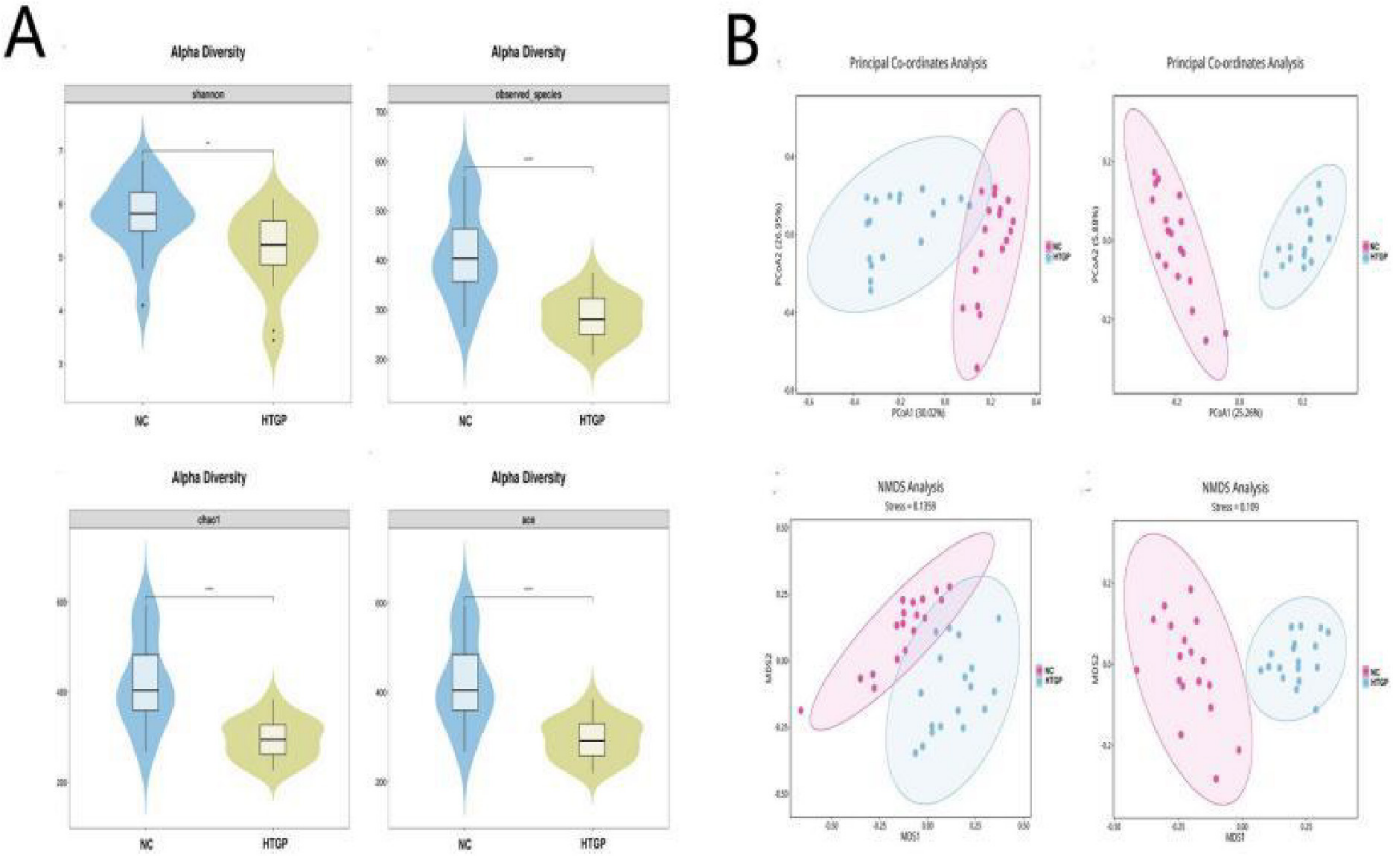
Species diversity analysis. **(A)** Alpha diversity of the intestinal flora. **(B)** Beta diversity of the intestinal flora. *P < 0.05, **P < 0.01, ****P < 0.0001.

Alpha diversity analysis using Shannon index, Observed Species, Chao1, and ACE indices revealed significant differences between the HTGP and NC groups (*P* < 0.01), indicating markedly lower species diversity in HTGP patients compared to healthy controls (Figure 1A). Regarding beta diversity, the two groups were significantly separated in coordinate space, indicating that the abundance distribution and phylogenetic relationships of the gut microbial communities in HTGP patients differed significantly from those in healthy controls. In summary, the gut microbiota structure of HTGP patients differed significantly from that of healthy volunteers, suggesting that the disease state exerts a significant impact on the gut microbial community (Figure 1B).

**FIGURE 2:**
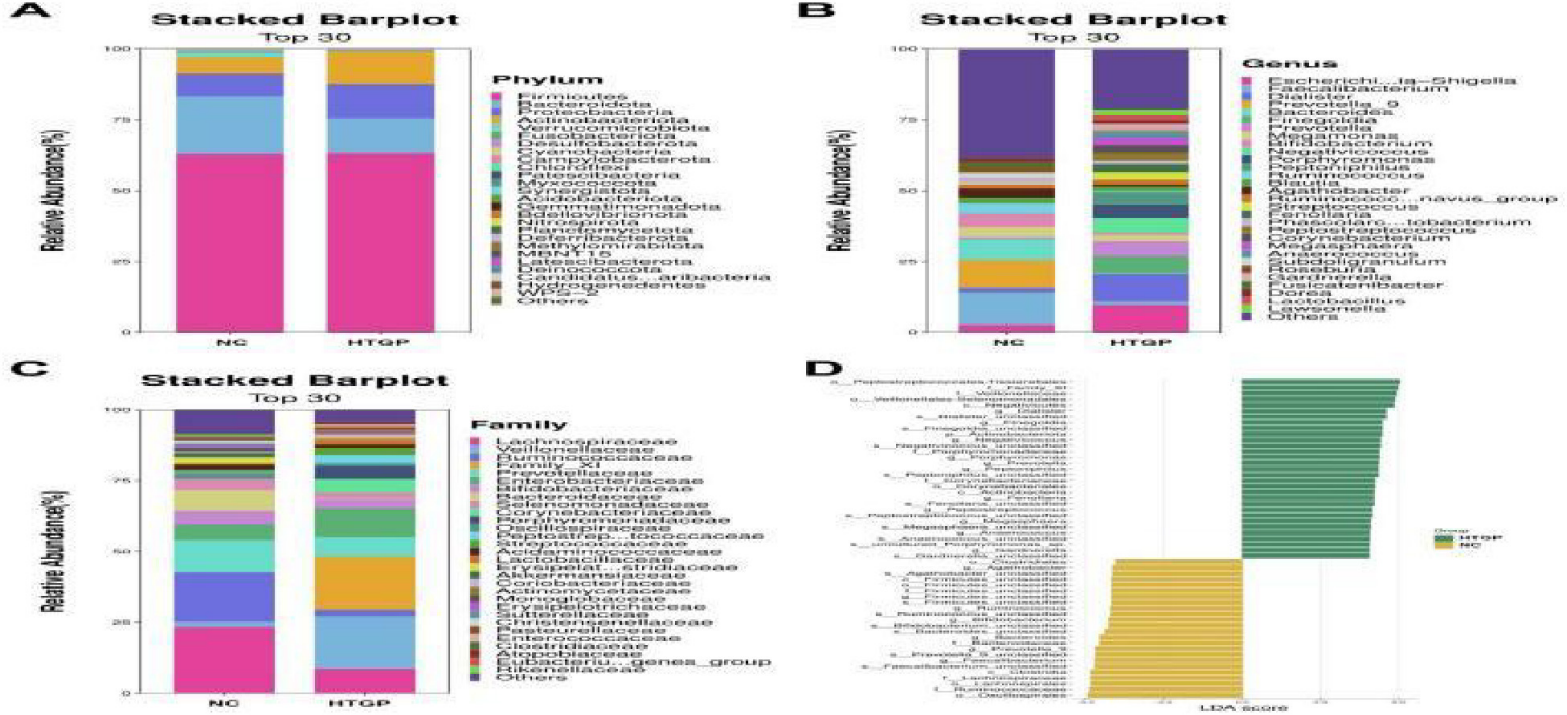
Relative abundance and LDA scores and functional annotations of major flora in both groups. **(A)** Relative abundance of major bacterial communities at the phylum level. **(B)** Relative abundance of major bacterial communities at the genus level. **(C)** Relative abundance of major bacterial communities at the family level. **(D)** LDA scores for different bacterial communities between the two groups.

**FIGURE 3:**
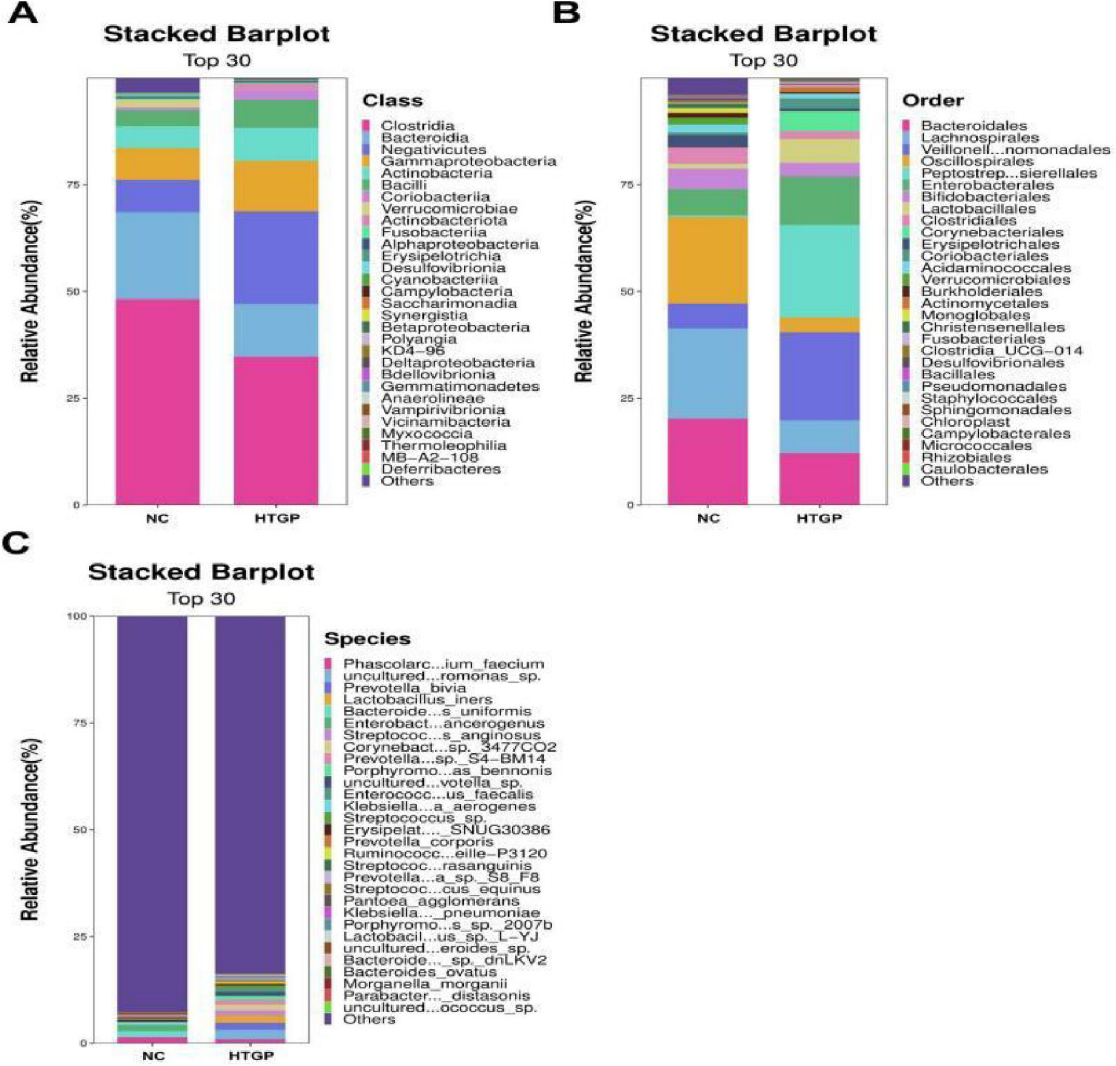
Relative abundance of major groups of bacteria at the level of class, order and species in the two groups. **(A)** Class level. **(B)** Order level. **(C)** Species level.

Based on species composition analysis, at the phylum level, both gut microbiota groups were dominated by Firmicutes, with relative abundances of 63.33% in the HTGP group and 63.07% in the NC group. However, compared to the NC group, the HTGP group exhibited significant dysbiosis: the relative abundance of Bacteroidetes was markedly reduced (12.31% vs. 20.44%), and that of Verrucomicrobia also decreased significantly (0.09% vs. 1.07%). Notably, the relative abundances of Proteobacteria and Actinobacteria significantly increased in the HTGP group, reaching 12.08% vs. 7.09% and 11.47% vs. 5.79%, respectively (Figure 2A). At the genus level. Results revealed increased relative abundances of Escherichia-Shigella (9.88% vs. 2.80%), Dialister (9.59% vs. 1.70%), Finegoldia (6.02% vs. 0.00%), and Prevotella (5.23% vs. 0.67%) were significantly higher than in the NC group. In contrast, the HTGP group exhibited significantly higher relative abundances of potentially probiotic genera: Faecalibacterium (1.30% vs. 11.36%), Prevotella_9 (0.01% vs. 9.86%), Bacteroides (0.31% vs. 7.32%), and Megamonas (2.15% vs. 3.64%) were significantly reduced in the HTGP group (Figure 2B). At the family level, compared with the NC group, the HTGP group showed significantly reduced relative abundances of potentially probiotic families including Lachnospiraceae, Ruminococcaceae, and Prevotellaceae, which possess potential probiotic functions, were significantly reduced in the HTGP group compared to the NC group. Conversely, the relative abundances of Veillonellaceae, Family XI, and Enterobacteriaceae, which are closely associated with inflammatory responses, were significantly increased (Figure 2C). Significant alterations in microbial composition were observed at the class, order, and species levels (Figures 3A–C).

**FIGURE 4:**
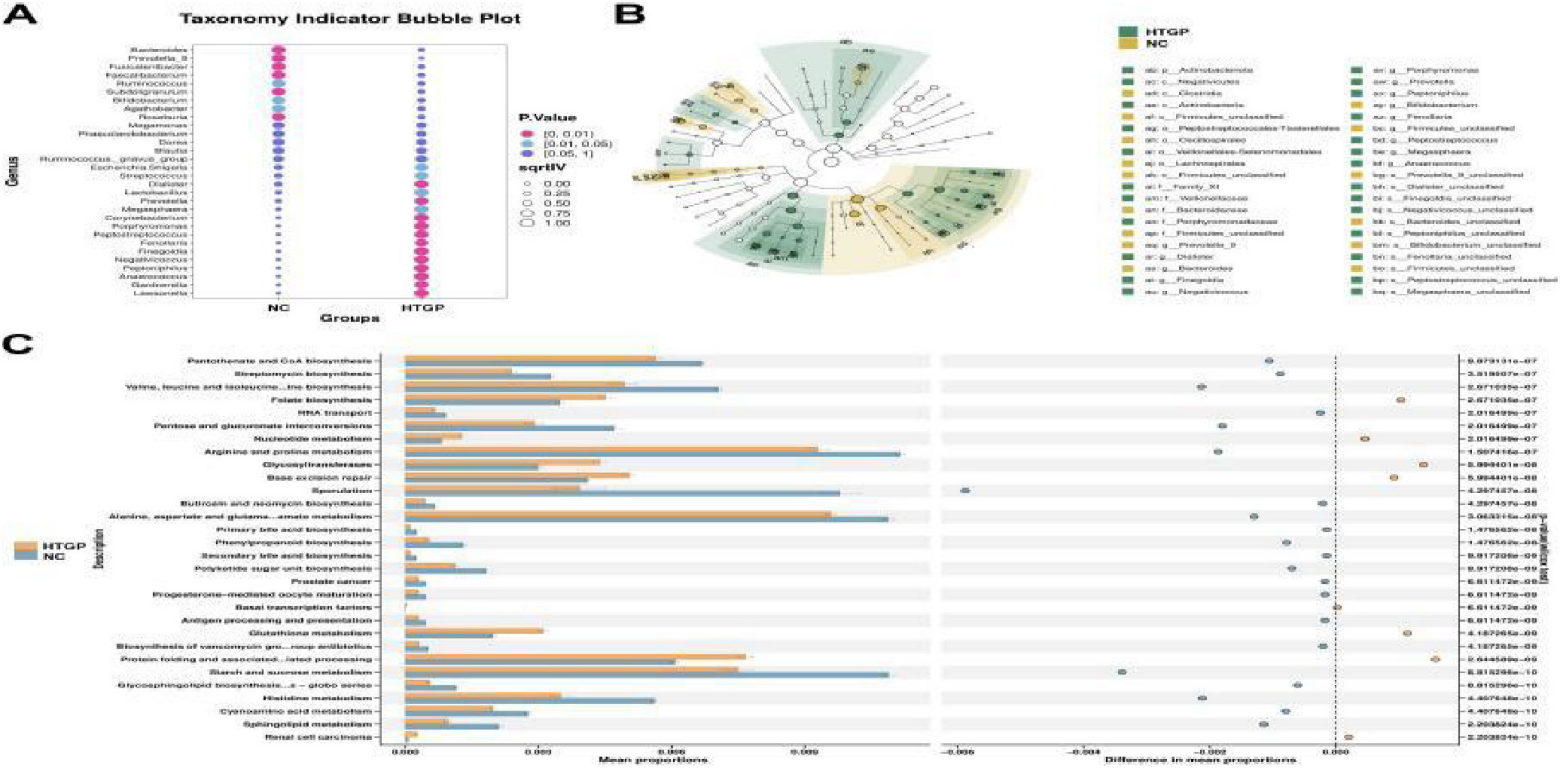
Differential analysis of intestinal flora and functional prediction results in the two groups. **(A)** Indicator species analysis plot, where dot size represents the sqrtIVt value. A higher value for a species indicates its greater potential as a biomarker for the treatment group. Colors indicate *P*-values; more significant *P*-values signify more reliable indicator values. **(B)** Phylogenetic tree of taxonomic groups: Yellow and green circles denote differences between the most abundant microbial communities. **(C)** STAMP differential analysis results for functional prediction across two groups based on the KEGG Level 3 database.

Based on intergroup differential species analysis using LEfSe, a total of 55 species were identified with significantly different distributions between the two groups (LDA score > 3.0, *P* < 0.05). In the HTGP group, the relative abundances of Peptostreptococcales-Tissierellales, Family XI, Veillonellaceae, Veillonellales-Selenomonadales, Negativicutes, and Dialister were significantly enriched (all LDA > 4.0, *P* < 0.05) (Figure 2D). Further validation via indicator species analysis revealed that in the HTGP group, the relative abundances of Escherichia-Shigella [sqrt (IV) > 0.75, *P* < 0.05], Dialister [sqrt (IV) > 0.75, *P* < 0.01], Finegoldia [sqrt (IV) > 0.75, *P* < 0.01], and Prevotella [sqrt (IV) > 0.75, *P* < 0.01] were significantly higher than those in the NC group. In contrast, the NC group exhibited significantly higher IVs for Faecalibacterium [sqrt (IV) > 0.75, *P* < 0.01], Prevotella_9 [sqrt (IV) > 0.75, *P* < 0.01], and Bacteroides [sqrt (IV) > 0.75, *P* < 0.01] were significantly higher than those in the HTGP group (Figure 4A). Significant differences in gut microbiota were observed between the HTGP and NC groups at the class, order, and species levels (Figure 4B).

Functional annotation analysis based on the KEGG Level 3 database revealed 30 significantly differentially activated pathways between the two groups (Figure 4C). Pathways related to inflammatory response, lipid metabolism, and carbohydrate metabolism were significantly enriched in the HTGP group, whereas pathways associated with short-chain fatty acid synthesis and intestinal barrier function maintenance showed higher activity in the NC group.

### 3.3 Serum SCFA metabolite analysis in HTGP patients

**FIGURE 5:**
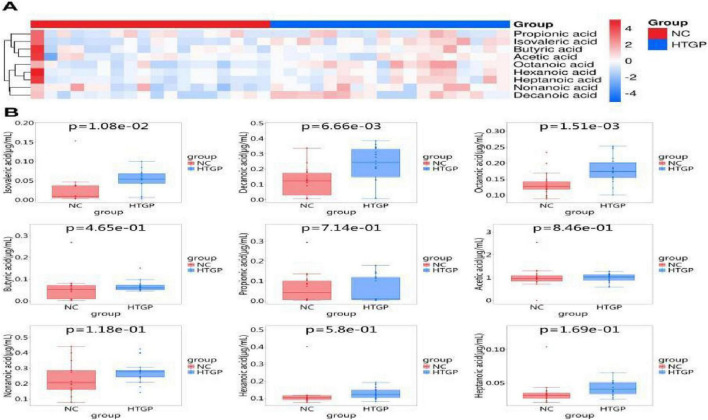
Cluster analysis and concentration distribution of serum SCFAs in two groups. **(A)** Hierarchical clustering analysis heatmap of SCFAs in the two serum groups. **(B)** Box plots of SCFA concentration distributions in the two serum groups.

Analysis of SCFA concentrations revealed differences in the distribution of 10 SCFAs between the HTGP and NC groups (Table 2). Concentrations of isovaleric acid (*P* = 0.011), decanoic acid (*P* = 0.007), and octanoic acid (*P* = 0.002) were significantly higher in the HTGP group compared to the NC group. Bioinformatics analysis identified the top nine significantly up-regulated and down-regulated differential metabolites in the HTGP group, visualized via a heatmap (Figure 5A). To further intuitively compare the distribution characteristics of each metabolite between groups, box plots were constructed to illustrate intergroup differences in metabolite concentrations and their dispersion (Figure 5B).

**TABLE 2:** Comparison of serum concentrations of SCFAs between the two groups (Mean ± SD).

SCFAs (μ g/mL)	HTGP group (*N* = 18)	NC group (*N* = 18)	*P*-value
Butyric acid	0.067 ± 0.025	0.053 ± 0.063	0.465
Propionic acid	0.052 ± 0.069	0.061 ± 0.079	0.714
Acetic acid	0.989 ± 0.195	1.013 ± 0.475	0.846
Non-anoic acid	0.277 ± 0.073	0.231 ± 0.097	0.118
Hexanoic acid	0.128 ± 0.031	0.118 ± 0.072	0.580
Heptanoic acid	0.042 ± 0.011	0.035 ± 0.018	0.169
Isobutyric acid	below LOD	below LOD	ND
Isovaleric acid	0.051 ± 0.028	0.019 ± 0.038	0.011
Valeric acid	below LOD	0.015 ± 0.056	0.266
Decanoic acid	0.224 ± 0.127	0.119 ± 0.094	0.007
Octanoic acid	0.180 ± 0.042	0.135 ± 0.036	0.002

*t*-tests.

**FIGURE 6:**
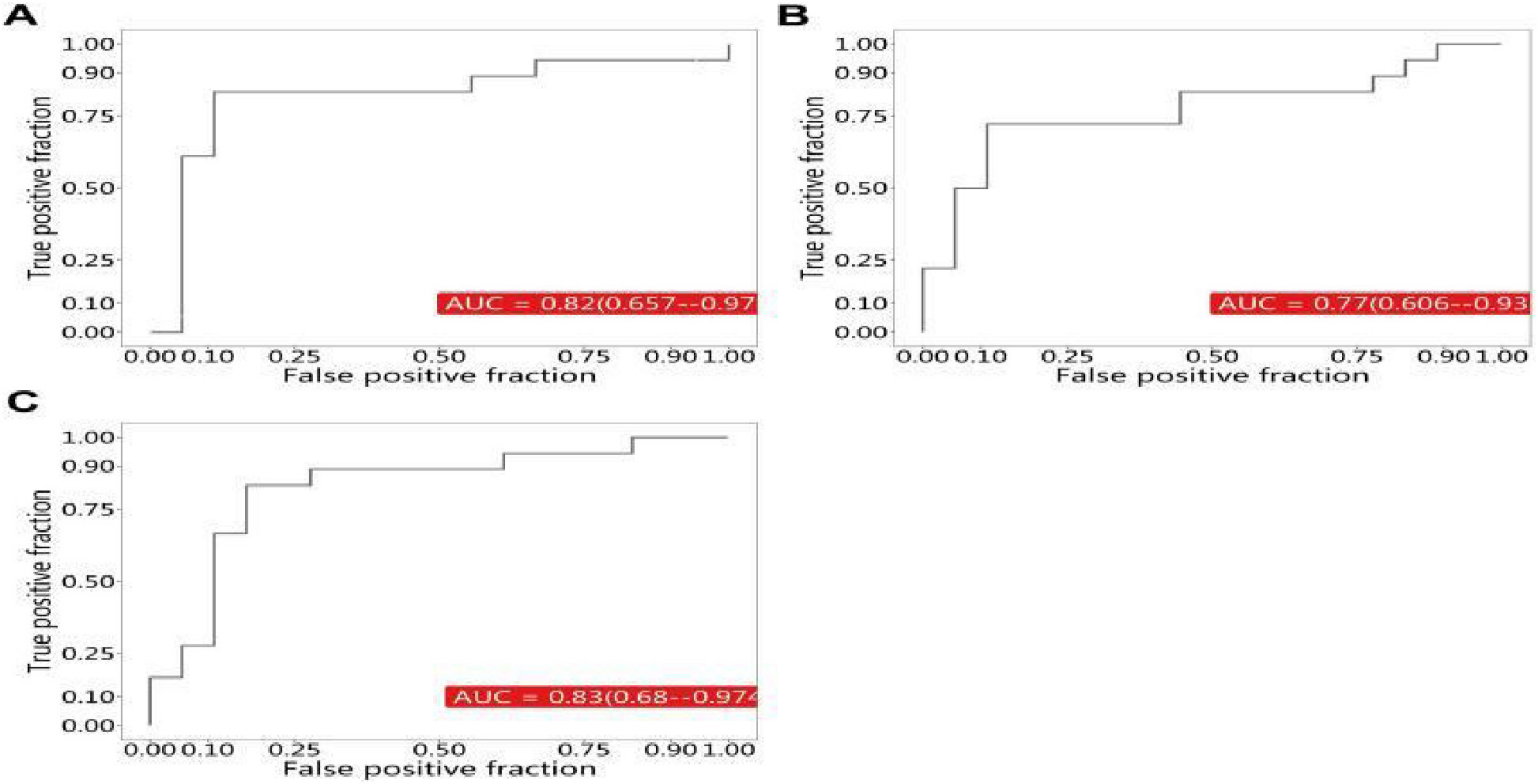
ROC curves of SCFAs in serum. **(A)** Isovaleric acid (NC vs. HTGP). **(B)** Capric acid (NC vs. HTGP). **(C)** Caprylic acid (NC vs. HTGP).

Based on the criteria of Variable Importance Projection (VIP) values > 1.0 and fold changes > 2.0 or < 0.5, SCFAs with significantly different concentrations were identified: isovaleric acid, capric acid, and caprylic acid (all *P* < 0.01). To evaluate the diagnostic and prognostic potential of these differential metabolites, receiver operating characteristic (ROC) curves were plotted and area under the curve (AUC) values calculated. Results showed AUC values of 0.820, 0.770, and 0.830 for isovaleric acid, capric acid, and caprylic acid, respectively, in distinguishing HTGP from NC groups (Figure 6).

### 3.4 Correlation analysis between gut microbiota and serum SCFA metabolites

Spearman correlation analysis was employed to systematically evaluate correlations between gut microbiota at the genus and family levels and differentially expressed metabolites. At the genus level (Table 3), analysis revealed that the abundance of Finegoldia was positively correlated with serum isovaleric acid levels (*r* = 0.519, *P* < 0.01); the abundance of Negativicoccus was positively correlated with serum isovaleric acid levels (*r* = 0.571, *P* < 0.001); and the abundance of Porphyromonas was positively correlated with serum isovaleric acid levels (*r* = 0.628, *P* < 0.001). In contrast, the abundance of Prevotella_9 showed a negative correlation with serum isovaleric acid levels (*r* = -0.503, *P* < 0.01).

**TABLE 3:** Correlation analysis of intestinal flora (genus level) with metabolites of SCFAs.

Intestinal flora (genus level)/SCFAs	Acetic acid	Butyric acid	Decanoic acid	Heptanoic acid	Hexanoic acid	Isovaleric acid	Non-anoic acid	Octanoic acid	Propionic acid
Bacteroides	0.061	−0.217	−0.413	−0.342	−0.342	−0.448	−0.218	−0.415	0.058
Bifidobacterium	0.003	0.010	0.003	−0.242	0.023	−0.149	−0.226	−0.220	0.129
Dialister	−0.079	0.150	0.140	0.102	0.139	0.186	−0.064	0.094	−0.142
Faecalibacterium	−0.168	−0.284	−0.307	−0.307	−0.275	−0.426*	−0.124	−0.336*	−0.053
Finegoldia	0.072	0.183	0.323	0.337*	0.382*	0.519**	0.236	0.482**	−0.056
Negativicoccus	0.090	0.191	0.321	0.268	0.303	0.571***	0.198	0.382*	−0.150
Peptoniphilus	−0.008	0.123	0.378*	0.339*	0.397*	0.497**	0.281	0.461**	−0.160
Porphyromonas	0.009	0.240	0.492**	0.401*	0.432**	0.628***	0.316	0.474**	−0.111
Prevotella	−0.042	0.195	0.336*	0.376*	0.340*	0.333*	0.135	0.471**	0.034
Prevotella_9	0.116	−0.331*	−0.453**	−0.338*	−0.403*	−0.503**	−0.139	−0.474**	−0.170

*P < 0.05, **P < 0.01, ***P < 0.001. Values represent Spearman correlation coefficients (*r*-values).

**FIGURE 7:**
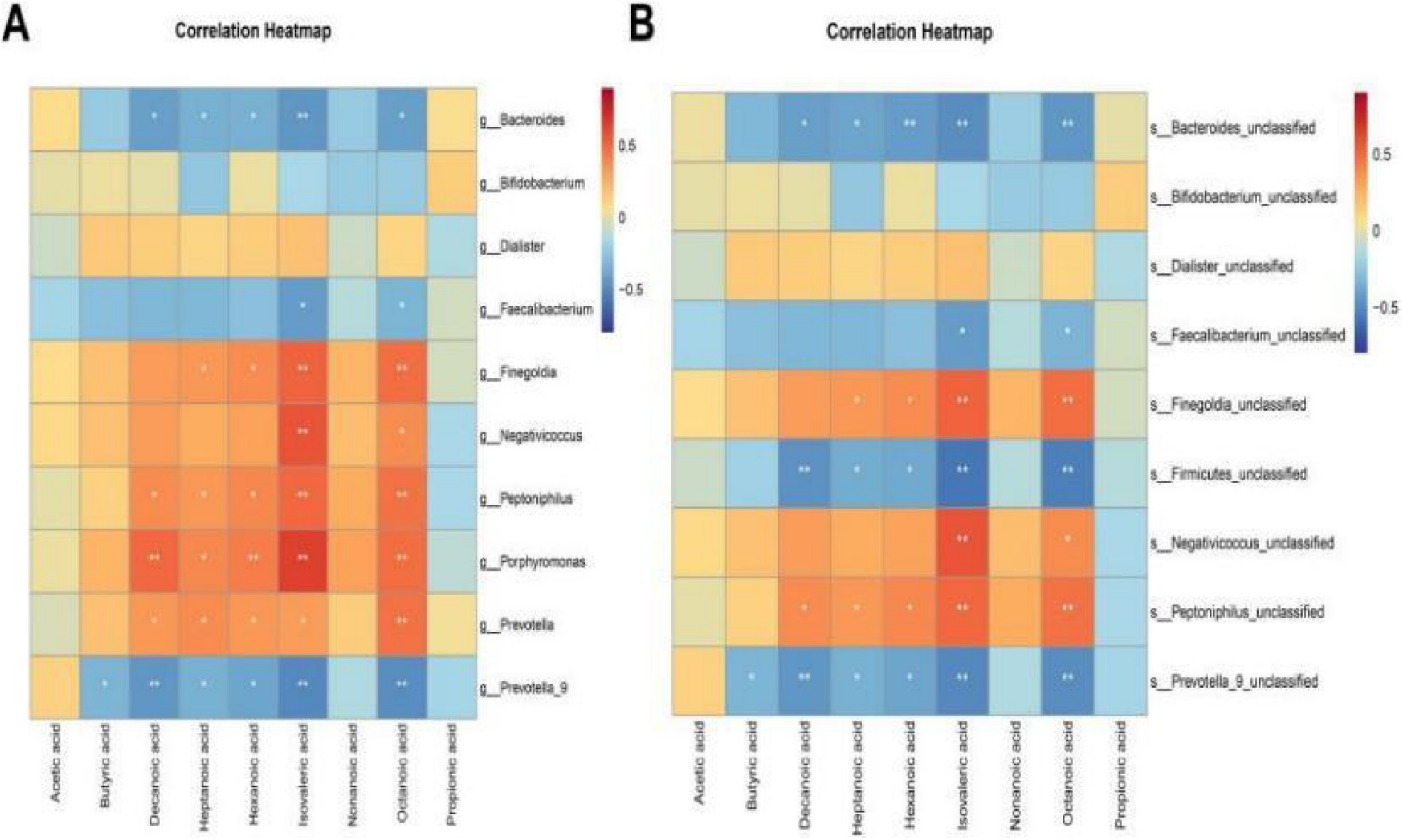
Heatmap of correlation analysis between intestinal flora and serum SCFAs metabolites. **(A)** Correlation between genus-level gut microbiota and serum SCFA metabolites. **(B)** Correlation between species-level gut microbiota and serum SCFA metabolites. The stronger the positive correlation, the closer it is to red; the stronger the negative correlation, the closer it is to blue; no correlation, the closer it is to yellow. *P < 0.05, **P < 0.01.

At the family level, correlation analysis between differential microbiota and differential SCFA metabolites (Table 4) revealed that the abundance of Finegoldia_unclassified was positively correlated with serum isovaleric acid levels (*r* = 0.519, *P* < 0.01); while the abundance of Negativicoccus_unclassified was positively correlated with serum isovaleric acid levels (*r* = 0.571, *P* < 0.001). In contrast, the abundance of unclassified Firmicutes (Firmicutes_unclassified) showed a negative correlation with serum isovaleric acid levels (*r* = -0.569, *P* < 0.001); The abundance of Prevotella_9_unclassified showed a negative correlation with serum isovaleric acid levels (*r* = -0.503, *P* < 0.01). Heatmaps visually presented the correlation analysis results between differential gut microbiota and serum SCFA metabolites (Figure 7).

**TABLE 4:** Correlation analysis of intestinal flora (species level) with metabolites of SCFAs.

Intestinal flora (species level)/SCFAs	Acetic acid	Butyric acid	Decanoic acid	Heptanoic acid	Hexanoic acid	Isovaleric acid	Non-anoic acid	Octanoic acid	Propionic acid
Bacteroides	0.014	−0.313	−0.420*	−0.398*	−0.436**	−0.483**	−0.212	−0.453**	−0.004
Bifidobacterium	0.003	0.010	0.003	−0.242	0.023	−0.149	−0.226	−0.220	0.129
Dialister	−0.079	0.150	0.140	0.102	0.139	0.186	−0.064	0.094	−0.142
Faecalibacterium	−0.168	−0.284	−0.307	−0.307	−0.275	−0.426*	−0.124	−0.336*	−0.053
Finegoldia	0.072	0.183	0.323	0.337*	0.382*	0.519**	0.236	0.482**	−0.056
Firmicutes	−0.072	−0.190	−0.462**	−0.370*	−0.375*	−0.569***	−0.118	−0.526**	−0.118
Negativicoccus	0.090	0.191	0.321	0.268	0.303	0.571***	0.198	0.382*	−0.150
Peptoniphilus	−0.008	0.123	0.378*	0.339*	0.397*	0.497**	0.281	0.461**	−0.160
Prevotella_9	0.116	−0.331*	−0.453**	−0.338*	−0.403*	−0.503**	−0.139	−0.474**	−0.170

*P < 0.05, **P < 0.01, ***P < 0.001. Values represent Spearman correlation coefficients (*r*-values).

### 3.5 Correlation analysis between gut microbiota, SCFA metabolites, and clinical case parameters

**FIGURE 8:**
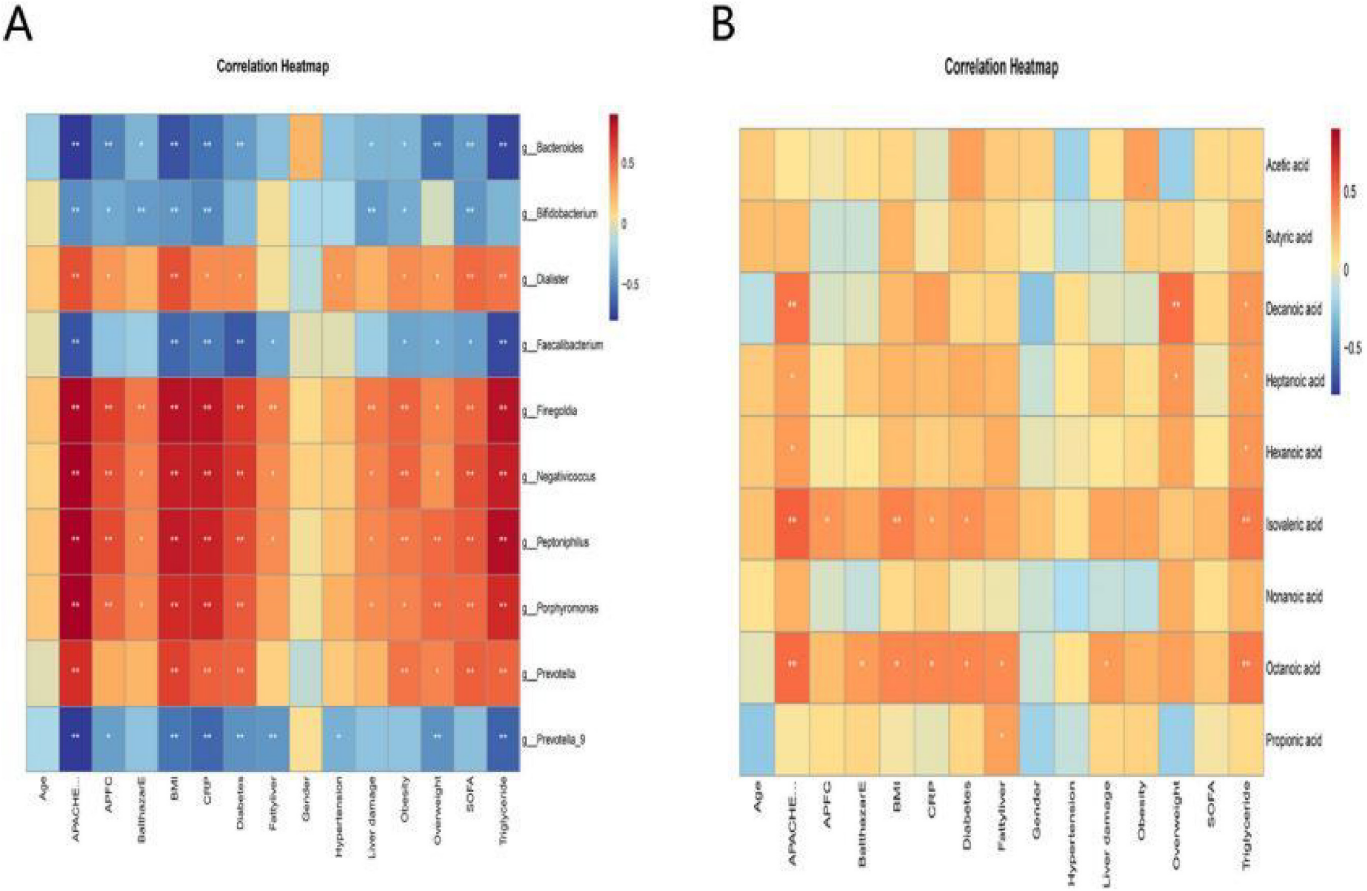
Heatmap of correlation analysis between clinical case parameters and gut microbiota as well as SCFA metabolites. **(A)** Heatmap for correlation analysis of intestinal flora with clinical case parameters. **(B)** Heatmap for correlation analysis of serum SCFAs metabolites with clinical case parameters. The stronger the positive correlation, the closer it is to red; the stronger the negative correlation, the closer it is to blue; no correlation, the closer it is to yellow. *P < 0.05, **P < 0.01.

Using Spearman correlation analysis, this study systematically evaluated correlations between genus-level gut microbiota and clinical case parameters (Table 5). Results showed that the abundance of Dialister was positively correlated with APACHE II scores and body mass index (BMI) (*r* > 0.5, *P* < 0.05); while the abundance of Finegoldia and Negativicoccus was positively correlated with APACHE II score, acute pancreatitis CT severity index (APFC), BMI, C-reactive protein (CRP) level, diabetes, obesity, Sequential Organ Failure Assessment (SOFA) score, and triglyceride level (*r* > 0.5, *P* < 0.05); the abundance of Peptoniphilus and Porphyromonas was positively correlated with APACHE II score, APFC, BMI, CRP level, diabetes, SOFA score, and triglyceride level (*r* > 0.5, *P* < 0.05). In contrast, Bacteroides abundance showed negative correlations with APACHE II scores, APFC, BMI, and CRP levels (*r* < -0.5, *P* < 0.05); Faecalibacterium abundance showed negative correlations with APACHE II scores, BMI, CRP levels, diabetes, and triglyceride levels (*r* < -0.5, *P* < 0.05). Heatmaps visually presented the correlation analysis results between differential gut microbiota and clinical case parameters (Figure 8A).

**TABLE 5:** Correlation analysis of intestinal flora with clinical case parameters.

Variables	Bacteroides	Bifidob	Dialiste	Faecalib acterium	Finegoldia	Negativ icoccus	Peptonip hilus	Porphyr omonas	Prev otella	Prevotella_9
Age	−0.200	0.007	0.158	0.004	0.179	0.120	0.173	0.178	−0.025	−0.150
APACHEII	−0.775***	−0.474**	0.589***	−0.695***	0.875***	0.886***	0.891***	0.887***	0.697	−0.769***
APFC	−0.521**	−0.373*	0.334*	−0.264	0.639***	0.585***	0.605***	0.514**	0.274	−0.421*
BalthazarE	−0.336*	−0.429**	0.259	−0.205	0.442**	0.406*	0.393*	0.381*	0.239	−0.263
BMI	−0.689***	−0.443**	0.585***	−0.618***	0.816***	0.766***	0.789***	0.719***	0.636	−0.563***
CRP	−0.594***	−0.504**	0.383*	−0.545**	0.799***	0.766***	0.757***	0.719***	0.526	−0.621***
Diabetes	−0.431**	−0.289	0.374*	−0.669***	0.643***	0.669***	0.594***	0.548**	0.518	−0.466**
Fatty liver	−0.273	0.036	0.036	−0.380*	0.429**	0.381*	0.368*	0.320	0.116	−0.451**
Gender	0.231	−0.161	−0.129	−0.027	0.092	0.111	0.040	0.034	−0.109	0.048
Hypertension	−0.253	−0.159	0.348*	−0.017	0.213	0.150	0.184	0.256	0.147	−0.339*
Liver damage	−0.336*	−0.429**	0.259	−0.205	0.442**	0.406*	0.393*	0.381*	0.239	−0.263
Obesity	−0.334*	−0.368*	0.368*	−0.388*	0.516**	0.508**	0.437**	0.403*	0.463	−0.262
Overweight	−0.560***	−0.055	0.334*	−0.380*	0.397*	0.368*	0.490**	0.503**	0.409	−0.468**
SOFA	−0.426*	−0.455**	0.490**	−0.413*	0.520**	0.577***	0.540**	0.502**	0.538	−0.273
Triglyceride	−0.744***	−0.328	0.460**	−0.725***	0.827***	0.777***	0.835***	0.726***	0.515	−0.632***

*P < 0.05, **P < 0.01, ***P < 0.001. Values represent Spearman correlation coefficients (*r*-values).

Concurrently, this study evaluated correlations between serum SCFA metabolites and clinical parameters (Table 6). Analysis revealed positive correlations between decanoate levels and APACHE II scores and obesity (*r* > 0.45, *P* < 0.05); isovaleric acid levels showed positive correlations with APACHE II scores, BMI, and triglyceride levels (*r* > 0.4, *P* < 0.05); octanoic acid levels exhibited positive correlations with APACHE II scores, BMI, CRP levels, diabetes, fatty liver, and triglyceride levels (*r* > 0.4, *P* < 0.05). Heatmaps visually presented the correlation analysis results between serum SCFA metabolites and clinical case parameters (Figure 8B).

**TABLE 6:** Correlation analysis of serum SCFAs metabolites with clinical case parameters.

Variables	Acetic acid	Butyric acid	Decanoic acid	Heptanoic acid	Hexanoic acid	Isovaleric acid	Non-anoic acid	Octanoic acid	Propionic acid
Age	0.169	0.217	−0.121	0.178	0.178	0.206	0.065	−0.026	−0.253
APACHEII	0.044	0.219	0.471**	0.332*	0.338*	0.536**	0.281	0.491**	0.031
APFC	0.013	−0.090	−0.045	0.039	0.039	0.354*	−0.045	0.225	0.077
BalthazarE	0.089	−0.081	−0.043	0.182	0.058	0.305	−0.112	0.336*	0.120
BMI	0.115	0.251	0.236	0.260	0.230	0.435**	0.103	0.404*	0.022
CRP	−0.037	0.012	0.326	0.235	0.146	0.345*	0.167	0.419*	−0.026
Diabetes	0.318	0.199	0.125	0.289	0.204	0.374*	0.017	0.408*	0.113
Fattyliver	0.165	0.122	0.143	0.251	0.287	0.316	−0.007	0.402*	0.330*
Gender	0.161	0.027	−0.274	−0.091	−0.016	0.210	−0.091	−0.091	−0.204
Hypertension	−0.213	−0.118	0.084	0.057	0.010	0.084	−0.152	0.071	−0.105
Liver damage	0.089	−0.081	−0.043	0.182	0.058	0.305	−0.112	0.336*	0.120
Obesity	0.321	0.159	−0.051	0.091	0.105	0.314	−0.125	0.267	0.139
Overweight	−0.229	0.148	0.479**	0.363*	0.311	0.189	0.299	0.328	−0.229
SOFA	0.094	0.034	0.113	−0.007	0.046	0.236	0.083	0.179	0.012
Triglyceride	0.124*	0.222	0.359*	0.339*	0.367*	0.442**	0.251	0.442**	0.104

*P < 0.05, **P < 0.01. Values represent Spearman correlation coefficients (*r*-values).

### 3.6 Butyrate alleviates pancreatic injury and reduce inflammatory factor release in HTGP rats

**FIGURE 9:**
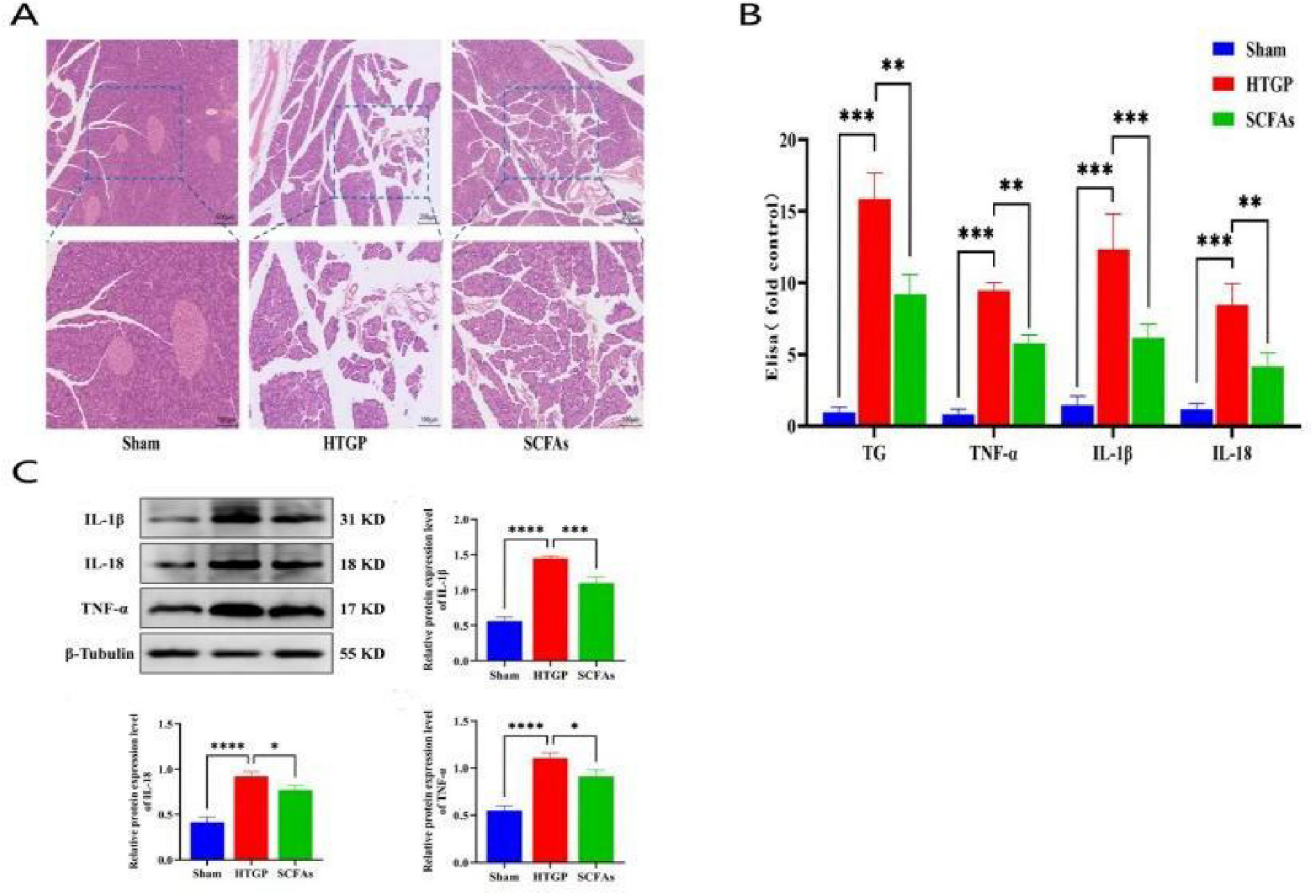
SCFAs Alleviate Pancreatic Injury and Reduce Inflammatory Factor Release in HTGP Rats. **(A)** Rat pancreas tissue H&E staining. **(B)** ELISA detection of serum TG and inflammatory cytokine levels. **(C)** Changes of inflammatory factor protein expression in pancreatic tissues of rats. *P < 0.05, **P < 0.01, ***P < 0.001

In the L-arginine-induced HTGP model, histopathological sections of SD rat pancreatic tissue stained with hematoxylin and eosin (HE) are shown in Figure 9A. The pancreas in the sham group exhibited tightly arranged acinar cells with abundant, uniformly stained cytoplasm, showing no vacuolar degeneration or necrosis. The stroma displayed no edema, hemorrhage, or fibrosis, with clear ductal and islet structures. In contrast, the pancreas in the HTGP group demonstrated significant pathological alterations, including disorganized acinar cell arrangement, vacuolar degeneration in some cells (visible vacuolar structures within cytoplasm), marked interstitial edema with abundant inflammatory cell infiltration; partial sloughing of ductal epithelial cells; uneven distribution of islet cells; and atrophy or necrosis in some islet cells. Additionally, rat serum ELISA results showed significantly higher levels of triglycerides (TG), inflammatory factors TNF-α, IL-1β, and IL-18 in the HTGP group compared to the Sham group (Figure 9B). Western blot analysis further confirmed that compared with the Sham group, the expression of TNF-α, IL-1β, and IL-18 in pancreatic cells was significantly upregulated in the HTGP group (all *P* < 0.01) (Figure 9C). Collectively, these results indicate that the HTGP model was successfully established, and the HTGP model group exhibited significant inflammatory responses and histopathological damage.

Following butyrate treatments, HE staining revealed significantly improved acinar cell arrangement, markedly reduced interstitial edema, and decreased inflammatory cell infiltration compared to the HTGP group (Figure 9A). Serum ELISA results indicated that TG levels and concentrations of inflammatory cytokines TNF-α, IL-1β, and IL-18 were significantly lower in the SCFAs + HTGP group than in the HTGP group (Figure 9B). Western blot analysis further confirmed that compared with the HTGP group, the expression of TNF-α, IL-1β, and IL-18 in pancreatic cells was significantly downregulated in the SCFAs + HTGP group (all *P* < 0.05) (Figure 9C). In summary, butyrate significantly alleviated L-arginine-induced histopathological damage in the pancreas of HTGP rats and suppressed the release of inflammatory cytokines, indicating the potential therapeutic role of butyrate in HTGP.

### 3.7 Butyrate modulates activation of the NF-κB signaling pathway and NLRP3 inflammasome

**FIGURE 10:**
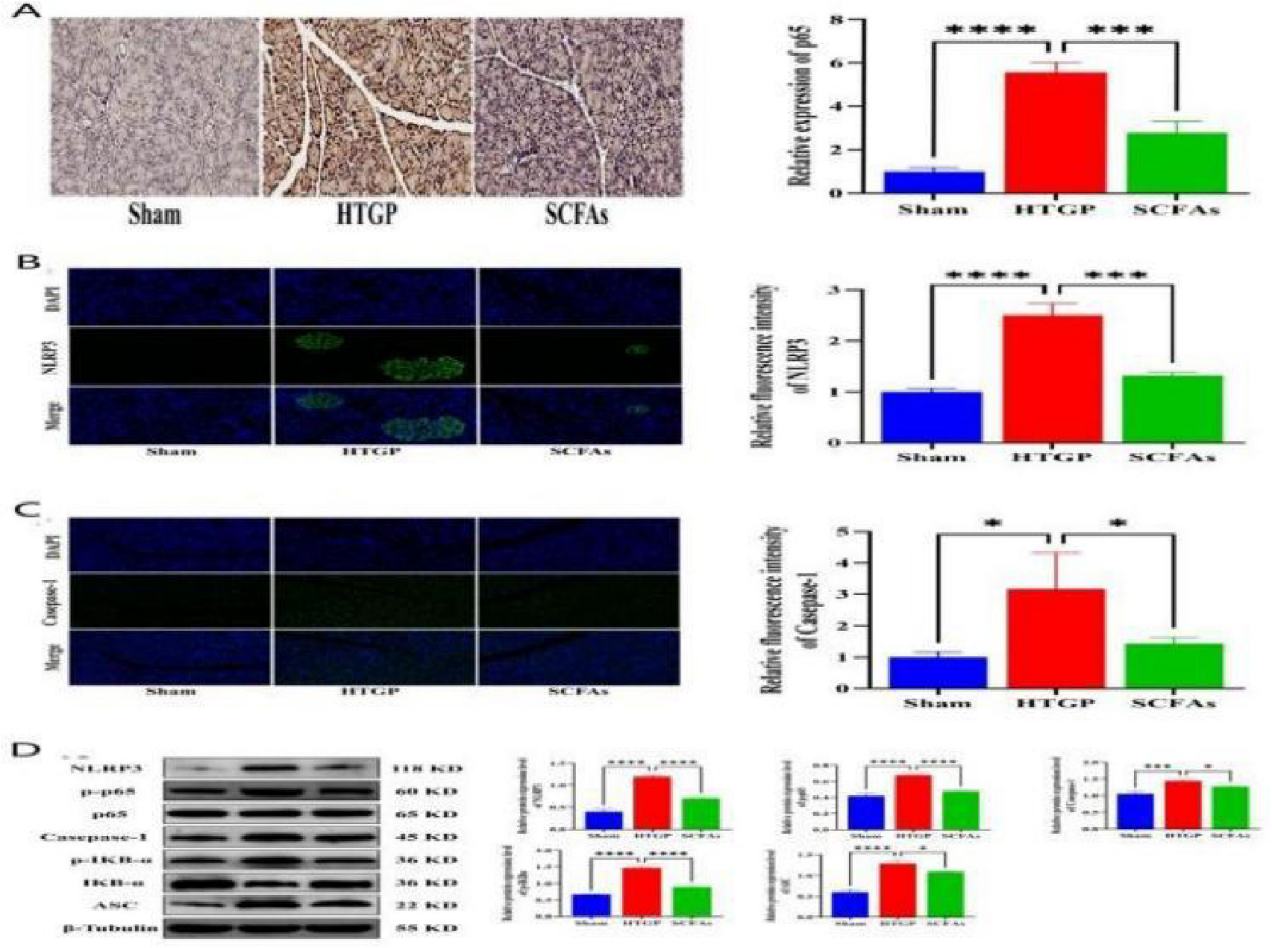
SCFAs Modulate Activation of the NF-κB Signaling Pathway and NLRP3 Inflammasome. **(A)** Immunohistochemistry of p65 in rat pancreatic tissue. **(B)** Immunofluorescence of NLRP3 in rat pancreatic tissue. **(C)** Immunofluorescence of NLRP3, p65, and Caspase-1 in rat pancreatic tissue. **(D)** Expression and relative quantification of NF- κ B signaling pathway and NLRP3 inflammatory vesicle-associated proteins. *P < 0.05, **P < 0.01, ***P < 0.001.

This study systematically evaluated the regulatory effects of butyrate on the NF-κB signaling pathway and NLRP3 inflammasome in HTGP rats using immunohistochemistry and immunofluorescence techniques. Immunohistochemical analysis revealed significantly enhanced nuclear translocation of p65 protein in HTGP-treated pancreatic tissue compared to the Sham group (*P* < 0.0001). In contrast, the SCFAs + HTGP group exhibited significantly reduced nuclear expression of p65 protein relative to the HTGP group (*P* < 0.001). Immunofluorescence detection revealed that compared with the Sham group, NLRP3 protein expression in islet tissue was significantly elevated in the HTGP group (*P* < 0.0001), and Caspase-1 protein expression on the cell membrane was significantly increased (*P* < 0.05). Following butyrate intervention, NLRP3 and Caspase-1 protein expression levels were significantly reduced, while these proteins were nearly absent in the Sham group (Figures 10A–C). Western blot analysis further confirmed that NLRP3, Caspase-1, ASC, p-p65, and p-IκBα protein expression levels in the HTGP group were significantly higher than in the Sham group (all *P* < 0.001); while the SCFAs + HTGP group exhibited significantly downregulated expression of these proteins compared to the HTGP group (Figure 10D). These findings indicate that butyrate effectively inhibit the activation of the NF-κB signaling pathway and NLRP3 inflammasome, thereby exerting their anti-inflammatory effects.

## 4 Discussion

The pathogenesis of HTGP is complex, involving multiple factors including metabolic abnormalities, inflammatory responses, and gut microbiota dysbiosis. In this study, HTGP patients exhibited significantly higher C-reactive protein levels and BMI compared to healthy controls, along with higher rates of diabetes and fatty liver disease. The relationship between obesity, inflammatory mediators, and pancreatitis has been confirmed by multiple studies (Khatua et al., 2017; Premkumar et al., 2015; Yan et al., 2024). Increased pancreatic fat deposition in obese patients may lead to pancreatic dysfunction and exacerbated inflammatory responses (Yang and McNabb-Baltar, 2020), consistent with our findings. This highlights that obesity management is a critical component in HTGP treatment.

Alterations in gut microbiota composition and diversity are closely associated with the onset and progression of pancreatic diseases (Brubaker et al., 2021; Frost et al., 2020). Particularly in the pathological process of HTGP, the interaction between the gut microbiome and the host plays a key regulatory role (Prukpitikul et al., 2024; Wang et al., 2024). This study identified significant alterations in the gut microbiota of HTGP patients. Beneficial bacterial groups such as Bacteroidetes and Verrucomicrobota were markedly reduced in the HTGP group, while potentially pathogenic groups like Proteobacteria and Actinobacteria showed significant proliferation. This aligns with previous findings showing higher abundances of potential pathogens (Klebsiella, Enterococcus, and Shigella) and lower abundances of beneficial bacteria (Faecalibacterium prausittis and Bacteroides uniformis) in HTGP patients (Li G. et al., 2023). Although the specific beneficial and potential pathogenic bacteria differed from our sequencing results, this may be attributed to geographical variations. Nevertheless, the trend of increased pathogenic bacteria and reduced beneficial bacteria in the gut microbiota of HTGP patients remains consistent. Furthermore, we observed a positive correlation between Faecalibacterium and diabetes as well as triglyceride levels. This suggests that gut dysbiosis is closely associated with the development of various metabolic disorders (Hiltunen et al., 2022). The altered gut microbiota in HTGP suggests that modulating the gut microbiome may represent a potential therapeutic approach for HTGP, and offering new insights for clinicians.

We analyzed differences in serum SCFAs between the two groups. In the HTGP group, serum concentrations of acetate, propionate, and valerate were lower than in the NC group, while serum concentrations of butyrate and isovalerate were higher than in the NC group. Reduced gut microbiota diversity leads to decreased SCFA synthesis, thereby affecting host metabolic health (Lee et al., 2021). In this study, gut microbiota alterations in HTGP patients may be a key factor contributing to altered SCFA levels, further increasing pancreatitis risk. Thus, modulating SCFA levels may represent a novel therapeutic approach.

To explore whether the differential microbiota and metabolites between groups were correlated and could serve as potential markers for HTGP, we analyzed correlations among differential gut microbes, metabolites, and clinical parameters. We found that the abundance of Fusobacterium, Gram-negative cocci, and Porphyromonas was significantly positively correlated with serum isovaleric acid levels. This suggests that potential pathogenic bacteria participate in the pathogenesis of HTGP by modulating the variety of SCFAs. In this study, the phylum Bacteroidetes was significantly reduced in the HTGP group, and the abundance of the genus Bacteroides showed significant negative correlations with APACHE II scores, BMI, and CRP levels. Furthermore, Bacteroides abundance was negatively correlated with the prevalence of fatty liver disease, obesity, and overweight individuals. Currently, the Bacteroidetes phylum has been demonstrated to mitigate disease progression by protecting the gut microbiota (Magne et al., 2020). Previous studies have shown that Bacteroidetes improves obesity and hepatic steatosis (López-Almela et al., 2021; Zou et al., 2024), consistent with our findings. This suggests that Bacteroidetes may serve as a potential biomarker in HTGP. Furthermore, serum isovaleric acid levels were positively correlated with triglycerides and APACHE II scores. This aligns with previous findings, confirming the crucial role of SCFAs in regulating host metabolic status (Hiltunen et al., 2022). It also suggests that SCFAs can assess disease severity and prognosis in HTGP patients. SCFAs may also reduce the risk of pancreatitis by alleviating inflammation through immune response regulation (Bin et al., 2025; Jarrett et al., 2021). In this study, although not statistically significant, acetate and propionate showed negative correlations with CRP. Thus, the altered microbiota and SCFAs in HTGP represent novel therapeutic targets.

To further explore the specific mechanisms of SCFAs in HTGP and their potential as therapeutic targets, this study successfully established an HTGP animal model. HTGP rats exhibited significant pathological damage and inflammatory responses in pancreatic tissues, consistent with descriptions of pancreatitis models in the literature, confirming the model’s stability and validity (Fei et al., 2020). SCFAs play crucial roles in regulating gut microbiota and suppressing inflammatory responses. We selected butyrate as the primary focus for the animal studies. Previous research has demonstrated that butyrate alleviates acute severe pancreatitis and necrotizing pancreatitis (Chen et al., 2024; van den Berg et al., 2021). However, our sequencing results revealed elevated butyrate levels in the HTGP group, seemingly contradicting prior studies. Therefore, we selected butyrate as the primary research target to validate its role and mechanism within the HTGP animal model. Our experimental results demonstrated that butyrate significantly ameliorate pathological changes in pancreatic tissue and reduce the release of inflammatory cytokines. This aligns with previous findings (Goraya et al., 2023; Hiltunen et al., 2022), suggesting the potential therapeutic value of butyrate in treating pancreatitis (Ibrahim et al., 2025; Li G. et al., 2023; Zhang et al., 2023). Our study revealed that NF-κB signaling pathway and NLRP3 inflammasome are activated in the HTGP model, which is closely associated with pancreatic injury and inflammatory responses. In the butyrate-treated group, NF-κB signaling pathway and NLRP3 inflammasome were significantly suppressed. NF-κB is activated may lead to upregulation of multiple proinflammatory factors, thereby exacerbating the inflammatory state in the pancreas (Chu et al., 2022; Dang et al., 2022; Gao et al., 2021; Jiang et al., 2025). Notably, under hypertriglyceridemic conditions, NLRP3 is activated may be associated with lipid metabolism disorders (Dang et al., 2022; Gao et al., 2021). Excessive triglycerides can induce intracellular oxidative stress, thereby activating NLRP3 inflammasomes and leading to pancreatic cell damage and exacerbated inflammatory responses (Al-Hawary et al., 2023; Choi et al., 2023). Previous studies have demonstrated that butyrate mitigates pancreatic injury by suppressing NF-κB activation and reducing inflammatory cytokine expression (Li G. et al., 2023; Yarmohammadi et al., 2021), consistent with our findings. This suggests butyrate may exert anti-inflammatory effects through regulating both the NF-κB signaling pathway and NLRP3 inflammasome.

Therefore, future research should further explore the potential application of SCFAs in pancreatitis and related diseases, particularly regarding their role in improving gut microbiota and regulating inflammatory responses. This could provide novel therapeutic strategies to enhance the prognosis of pancreatitis patients. This study has certain limitations. First, in experimental design, only butyrate was selected as the intervention group for validation. Based on preliminary metabolomics analysis, SCFAs such as isovaleric acid also exhibited significant alterations in the serum of HTGP patients. Consequently, subsequent studies should consider incorporating additional SCFAs into systematic intervention experiments to comprehensively evaluate the protective effects of different SCFAs on HTGP and their underlying molecular mechanisms. This refinement will contribute to a more accurate elucidation of the therapeutic value of SCFAs in HTGP. Second, due to the pathophysiological characteristics of pancreatitis (Bejjani et al., 2025), patients in the HTGP group experienced difficulty defecating, so we collected stool samples via anal swabs. This will lead to methodological mismatch. Although previous studies have indicated that the microbiomes of stool and rectal swabs from the same subject are highly similar, making these two sampling methods interchangeable (Bassis et al., 2017), it is preferable to standardize the collection method for both groups to enhance the accuracy of the results. Third, due to the small sample size, the power of multiple correlation tests is limited. In subsequent research, larger sample sizes should be employed for testing as needed. Finally, building on our findings, future studies could further explore the specific mechanisms by which SCFAs modulate the NF-κB signaling pathway and NLRP3 inflammasome. A deeper understanding of the interactions between the NF-κB signaling pathway and NLRP3 inflammasome may provide novel therapeutic targets and strategies for hypertriglyceridemic pancreatitis. Furthermore, considering the safety profile and potential clinical applications of SCFAs, future clinical trials will be essential to validate their efficacy and feasibility in the treatment of pancreatitis (Chen et al., 2023; Wu et al., 2022; Yu et al., 2022).

## Data Availability

The original contributions presented in this study are included in this article/Supplementary material, further inquiries can be directed to the corresponding author.
