# Supplementary material for: The mechanism of short-chain fatty acid in hypertriglyceridemic pancreatitis
Source: Front Microbiol. 2026 Feb 3;16:1714013. doi: 10.3389/fmicb.2025.1714013 (PMC12910481; doi:10.3389/fmicb.2025.1714013)
Supplement: Supplementary file 1 [file Table_1.pdf]

Tab.1 Demographic and clinical characteristics of the two groups

**Variables**	**HTGP group (N=18)**	**NC group (N=18)**	***P* value**
Age (years), mean (SD)	38.8（9.4）	37.4（10.6）	0.680
Male, n (%)	10（55.6）	10（55.6）	1.000
BMI (kg/m2), mean (SD)	29.0（3.0）	21.2 (2.0)	<0.001
Overweight (BMI 25–29.9 kg/m2), n (%)	10 (55.6)	1 (5.6)	0.003
Obesity (BMI ≥ 30 kg/m2), n (%)	7（38.9）	0（0.0）	0.008
Smoking, n (%)	9（50.0）	11（61.1）	0.738
Drinking, n (%)	14（77.8）	16（88.9）	0.658
Comorbid abnormalities, n (%)			
Hypertension	5（27.8）	2（11.1）	0.402
Diabetes	12（66.7）	0（0.0）	<0.001
Fatty liver	6（33.3）	0（0.0）	0.019
Laboratory examinations			
Triglyceride (mmol/L), median (IQR)	39.1（21.1, 49.4）	1.1（0.8, 1.4）	<0.001
CRP (mg/L), median (IQR)	29.4（7.1, 87.5）	2.2（1.9, 3.2）	<0.001
APACHE II, median (IQR)	2.0（2.0, 2.5）	0.0（0.0, 0.0）	<0.001
SOFA score, median (IQR)	0.0（0.0, 1.0）	0.0（0.0, 0.0）	0.004
Balthazar score E, n (%)	5（27.8）	0（0.0）	0.045
Local complications, n (%)			
APFC	8（44.4）	0（0.0）	0.003
ANC	1（5.6）	0（0.0）	1.000
Infected necrosis	1（5.6）	0（0.0）	1.000
Systematic complication, n (%)			
SIRS	4（22.2）	0（0.0）	0.104
AKI	1（5.6）	0（0.0）	1.000
Liver damage	5（27.8）	0（0.0）	0.045
Outcome			
Hospital stay (days), median (IQR)	9.5（6.8, 12.5）	0.0（0.0, 0.0）	<0.001
